# A systematic review and meta-analysis to identify vertical limits for a high-dose opioid therapy

**DOI:** 10.1186/s42466-025-00437-5

**Published:** 2025-10-17

**Authors:** Franziska Dickmann, Julia Stingl, Angelika Lampert, Martin Mücke, Vera Peuckmann-Post, Walter Magerl, Roman Rolke, Sascha Weber

**Affiliations:** 1 Department of Palliative Medicine, Medical Faculty, RWTH Aachen University, Pauwelsstraße 30, 52074 Aachen, Germany; 2 Department of Psychiatry, Psychotherapy and Psychosomatics, Medical Faculty, RWTH Aachen University, Aachen, Germany; 3 Institute of Clinical Pharmacology, Medical Faculty University Heidelberg, Heidelberg, Germany; 4 Institute of Neurophysiology, Medical Faculty, RWTH Aachen University, Aachen, Germany; 5 Institute for Digitalization and General Medicine, Medical Faculty, RWTH Aachen University, Aachen, Germany; 6 Center for Rare Diseases Aachen (ZSEA), Medical Faculty, RWTH Aachen University, Aachen, Germany; 7 Department of Anesthesiology, Medical Faculty, RWTH Aachen University, Aachen, Germany; 8 Institute for Occupational, Social and Environmental Medicine, Medical Faculty RWTH, Aachen University, Aachen, Germany; 9 Chair of Neurophysiology, Mannheim Center for Translational Neuroscience, Ruprecht Karls University Heidelberg, Mannheim, Germany; 10 Scientific Center for Neuropathic pain Aachen SCN AACHEN, Medical Faculty, RWTH Aachen University, Aachen, Germany

**Keywords:** Chronic pain, Cancer pain, Non-cancer pain, Opioids, High-dose, Threshold

## Abstract

**Background:**

Chronic pain represents the defining and quality-of-life limiting feature in patients with cancer pain (CP) or chronic non-cancer pain (CNCP) and is often treated with opioids. Over time, opioid use is frequently accompanied by necessity of an increasing dose due to pharmacological tolerance and progress of the underlying diseases. The potential side effects were found to correlate with accelerating doses. More recently, the opioid crisis in the United States has drawn attention to the adverse effects and toxicities. Until today it is unclear what high-dose opioid therapy is and guidelines are inconsistent regarding an evidence-based threshold.

**Objectives:**

This systematic review and meta-analysis aim to determine a threshold for high-dose opioid therapy. A systematic literature search was conducted in 4 databases from earliest publication available until May 2025. Studies were eligible if participants with CP or CNCP were able to self-titrate their opioid dosage to reach a sufficient pain relief.

**Methods:**

4305 records were screened. Nineteen included studies with a total of 3111 participants investigating eight different opioids were included. The studies were assessed for risk of bias. Results were synthesised as oral morphine equivalents (OMEs).

**Results:**

The meta-analysis found a weighted mean of 74.7 mg OME per day and the 97.5% percentile corresponded to about 138 mg/d (range 134–139 mg/d) as a “high dose”. In CNCP the limit was 78 mg/d (range 74–78 mg/d), whereas in CP it reached 288 mg/d (range 280–289 mg/d; *p* < 0.01).

**Conclusion:**

Despite the overall moderate risk of bias of the included studies and the heterogeneity in underlying pain conditions, the reference range of typically prescribed dosages in a broad study population could be investigated. These systematically derived thresholds may enhance physicians’ awareness in carefully tailoring opioid treatments and thereby contribute to improved pharmacotherapy safety.

**PROSPERO Identifier:**

CRD42020219256.

**Supplementary Information:**

The online version contains supplementary material available at 10.1186/s42466-025-00437-5.

## Introduction

Pain is a debilitating symptom in many various conditions and one of the most common reasons for patients to visit a doctor (Moore et al. [1]). Its definition has recently been revised by the International Association for the Study of Pain (IASP) to “an unpleasant sensory and emotional experience associated with actual or potential tissue damage”. The discrimination of acute pain with an important protective function for the human body versus chronic pain, that increasingly is understood as a condition on its own, is vital (Cohen et al. [2]).

At a pathophysiological level, a distinction is typically made between nociceptive and neuropathic pain (Becker et al. [3]; Kosek et al. [4]). Besides, in chronic pain there is a third component, called nociplastic pain, depicting the mechanisms that cause altered pain processing and modulation (Fitzcharles et al. [5]; Woolf [6]). The term “mixed pain” is likewise used to subsume chronic pain states with an overlap of all three categories (Freynhagen et al. [7]). The underlying mechanisms of chronic pain are not yet fully understood, which emerges as problematic considering the desirability of treatments according to the driving causes rather than symptoms (Kosek et al. [4]).

Due to the ageing population, the prevalence of cancer and chronic diseases associated with pain is increasing (Dahlhamer et al. [8]). Almost 30% of the adult population in the European Union (Leadley et al. [9]), more than 40% in the UK (Fayaz et al. [10]) and about 20% in the United States (Dahlhamer et al. [8]) are suffering from chronic pain, defined by the World Health Organization (WHO) as a condition persisting for more than 3 months (WHO [11]). In Denmark, epidemiological studies have even shown an increasing prevalence of chronic pain in the general population from 2000 to 2017 (Ekholm et al. [12]). Chronic pain impairs quality of life by compromising daily activities, social life, sleep quality, ability to work and therefore results in an enormous burden not only for the individual, but on society (Disease [13]; Fredheim et al. [14]).

According to the formerly used WHO analgesic ladder established in 1986, opioids played a major role in severe pain and have been well established for the treatment of cancer pain (CP) (Wiffen et al. [15]; World Health Organization [16]). Opioid agonists decrease the perception of pain and increase tolerance to painful stimuli through attachment to µ-opioid receptors. As the prevalence of chronic pain increases, opioids are likewise prescribed for the treatment of chronic non-cancer pain (CNCP) (Dowell et al. [17]) even though the benefits, especially in long-term use, are critically discussed (Busse et al. [18]; Chou et al. [19]; Häuser et al. [20]). The umbrella term CNCP covers chronic neuropathic pain for which treatment with strong opioids is accepted if first or second-line treatment fails (Finnerup et al. [21]; Sommer et al. [22]).

The pharmacological mechanism of opioids induces some amount of tolerance over time (Kosten & George [23]) and eventuates in diminished efficacy. Therefore the development of physical dependence might occur (Dowell et al. [17]) which in turn leads to increasing dosage (Spooner et al. [24]) and possibly problematic patterns of use in selected patients (Musich et al. [25]; Zutler & Holty [26]). This phenomenon occurs even if opioids have been initially prescribed for the treatment of severe pain (Kosten & George [23]) and in accordance with guidelines (Els et al. [27]). “Concomitant with this practice change, rates of fatal opioid overdose have increased (Dunn et al. [28])”, as Dunn et al. and Gomes et al. identified (Gomes et al. [29]). Lately, the opioid epidemic in the United States has drawn broader attention to the safety of opioids (National Institute on Drug Abuse [ [30]]).

Numerous studies have investigated the safety and tolerability of opioids and found increased risk for gastrointestinal and central nervous system side effects (Els et al. [27]). Common adverse effects include nausea, obstipation, sedation, dizziness up to falls and fractures particularly in the elderly, causing opioid-related emergency room visits or hospitalizations (Solomon et al. [31]). Especially the mechanism behind the increased risk for cardiovascular events and myocardial infarction is not fully understood yet (Solomon et al. [31]). A recent study found evidence of how oxycodone could affect cardiac sodium channels and potentially may lead to cardiac arrythmia when extremely high doses were administered (Meents et al. [32]). It has been noticed that adverse events are associated particularly with long-term prescription and high dosages (Els et al. [27]; Nadeau & Lawhern [33]).

Although the term ‘high-dose opioid therapy’ is widely used, there is no generally approved definition for a threshold (Musich et al. [25]). Numerous different dosages of 50 mg up to > 200 mg oral morphine equivalent (OME) per day are mentioned in the literature (Els et al. [34]; Kahan et al. [35]; Manchikanti et al. [36]; Musich et al. [25]; National Committee for Quality Assurance [37]; Spooner et al. [24]), but without giving a sufficiently justified method for setting this cut-off. There have been attempts to incorporate the correlation of higher dosages with increase in adverse events (Busse et al. [38]; Häuser et al. [39]) as well as duration of the treatment and polypharmacy (Musich et al. [25]). Due to the lack of a systematic approach to define a threshold for high-dose opioid therapy, none of the dosages has been established as universally applicable so far.

The recommendation of a threshold could provide guidance for physicians, may improve drug therapy safety and prevent harmful and unintended side effects. Therefore, a mathematical model could help to identify a threshold, which subsequently could be clinical investigated.

The aim of this systematic review and meta-analysis is to define a threshold for high-dose opioid therapy in patients with CP or CNCP based on a comparison to the reference range of typically prescribed opioid dosages found in the published literature. Secondary research objectives include the analysis of subgroups such as CP versus CNCP, strong opioid-naive versus experienced, based on age, baseline pain, decrease in pain and on the opioid administered.

## Methods

This review was undertaken in accordance with the PRISMA 2020 statement (Preferred Reporting Items for Systematic Reviews and Meta-Analyses) (Page et al. [40]) and following the recommendations of the Cochrane Collaboration (Lasserson et al. [41]). The PRISMA 2020 checklist can be found in the appendix. The protocol was disclosed before initiation of the literature search (PROSPERO Identifier CRD42020219256). Renal and hepatic impairment were determined as additional exclusion criteria since both organs play an important role in the metabolism of opioids.

### Search strategy

The following databases were searched from earliest publication available to May 2025: MEDLINE, EMBASE, CENTRAL (Cochrane Central Register of Controlled Trials) and PsycINFO. Combined terms were generated for “chronic pain” and “titrated opioid therapy” (see appendix for the specific search strings for each database). Sufficient sensitivity of the search strategy was ensured by checking for key papers already known to the authors. The reference lists of included studies were also checked to identify further studies of interest.

### Inclusion and exclusion criteria

Studies meeting all the following criteria were included:

1. Participants with chronic pain, defined as pain lasting for more than 3 months referring to the WHO (WHO [11]).
2. Any study design in which participants receive opioids for moderate to severe pain (“Guideline on the clinical development of medicinal products intended for the treatment of pain,“ [ [42]]) allocated either to a treatment group or a control group.
3. Patients were able to self-titrate the dosage of their opioid prescription to reach a sufficient pain relief, defined as at least 30% reduction in intensity compared with baseline (“Guideline on the clinical development of medicinal products intended for the treatment of pain,“ [ [42]]) or pain decrease to ≤ 3 on a numerical rating scale from 0 to 10.
4. Full text was available in English or German.

Studies were excluded if:

1. Pain occurred in an acute setting, e.g. fractures, postoperative pain, procedural pain, renal colic.
2. Opioid prescription was not carried out according to any guideline, e.g. indications like headache, functional disorder, fibromyalgia syndrome (Häuser et al. [20]).
3. Opioids were prescribed for indications other than pain, e.g. to treat dyspnea.
4. Opioids were prescribed as maintenance therapy or participants had a history of opioid dependence.
5. The participants showed renal or hepatic impairment.
6. The study was an animal study or meta-analysis.

The records were exported to EndNote X9 and duplicates were removed, followed by two researchers (FD, SW) independently screening titles and abstracts. Full texts were obtained for the selected studies and again examined by the two researchers independently. Authors of studies missing data for clarification of eligibility criteria were contacted via email or “researchgate.net”. Studies for which full text was not available were excluded. Any disagreement was solved by discussion until a final consensus was reached.

### Data extraction

Thereupon data on author, country of origin, study design, experimental unit, treatment duration, participant characteristics, pain condition, type of pain, opioid administration, opioid-naivety, co-medication, opioid dose, additional rescue medication, pain score at start and endpoint of the studies were extracted using a standardised data extraction form. The template was designed based on The Cochrane Developmental, Psychosocial and Learning Problems Review Group (The Cochrane Developmental Psychosocial and Learning Problems Review Group [43]) and implementing MECIR standards (Methodological Expectations of Cochrane Intervention Reviews) (Lasserson et al. [41]). Authors of studies missing particular outcome data were contacted via email or researchgate.net. Microsoft Excel was used to manage and analyse the extracted data. The spreadsheet was checked by a second reviewer.

### Data synthesis

The effect sizes for metric data (e.g. number of participants, age, opioid dosage) were expressed as mean, standard deviation (SD) and 95% confidence intervals. For one study missing the SD for the opioid dosage but providing a minimum and maximum, the SD was roughly estimated using the Range Rule of Thumb, which states that the range is about four times the SD (Ramirez & Cox [44]). Ordinal outcomes (e.g. pain rating) were reported as minimum, maximum and median. Pain ratings were reported on different scales and therefore initially combined into a standardized 0 to 10 scale (Hawker et al. [45]).

To compare the opioid dosage, a conversion of different opioids and routes of administration into an OME dose per day was necessary. Sources from the CDC guideline (Dowell et al. [46]); the „synthesis of oral morphine equivalents for opioid utilisation“ by Nielsen et al. (Nielsen et al. [47]), the “National Institutes of Health (NIH) Helping to End Addiction Long-term (HEAL) morphine milligram equivalent calculator” (Adams et al. [48]) and Von Korff’s et al. CONSORT classification of morphine equivalent conversion factors (Von Korff et al. [49]) were used and cross-referenced with the German guidelines for long-term opioid therapy (Häuser et al. [20]; Meißner & Radbruch [50]) and the conversion rates from the respective studies, if given, or with the respective prescribing information, if the exact preparation was mentioned. The same conversion rate was used for oxycodone and the combination preparation oxycodone/naloxone; therefore, the term “oxycodone” will be referred to collectively in the following.

The unit of measurement for all dose data was milligrams, the transdermal administered drugs buprenorphine and fentanyl were translated from µg per hour to mg per day.

An OME for each study group was calculated from the sum of the average daily dose at study endpoint, respectively the point in time at which sufficient titration for pain control was achieved, and the daily rescue dose, if provided. An OME of the SD was likewise calculated for each study.

A weighted mean was calculated for the entirety of all studies and different subgroups. The subgroups were formed based on underlying pain condition, opioid administered, opioid-naivety, decrease in pain and average age of the participants. To differentiate by age, baseline pain and decrease in pain, two groups each were formed, distributed as evenly as possible among the total number of participants. The cut-off value for groups of different ages was also consistent with the average age across all studies.

Differences between subgroups were reported descriptively. All calculations were performed using Microsoft Excel.

### Meta-analysis

**Fig. 1:**
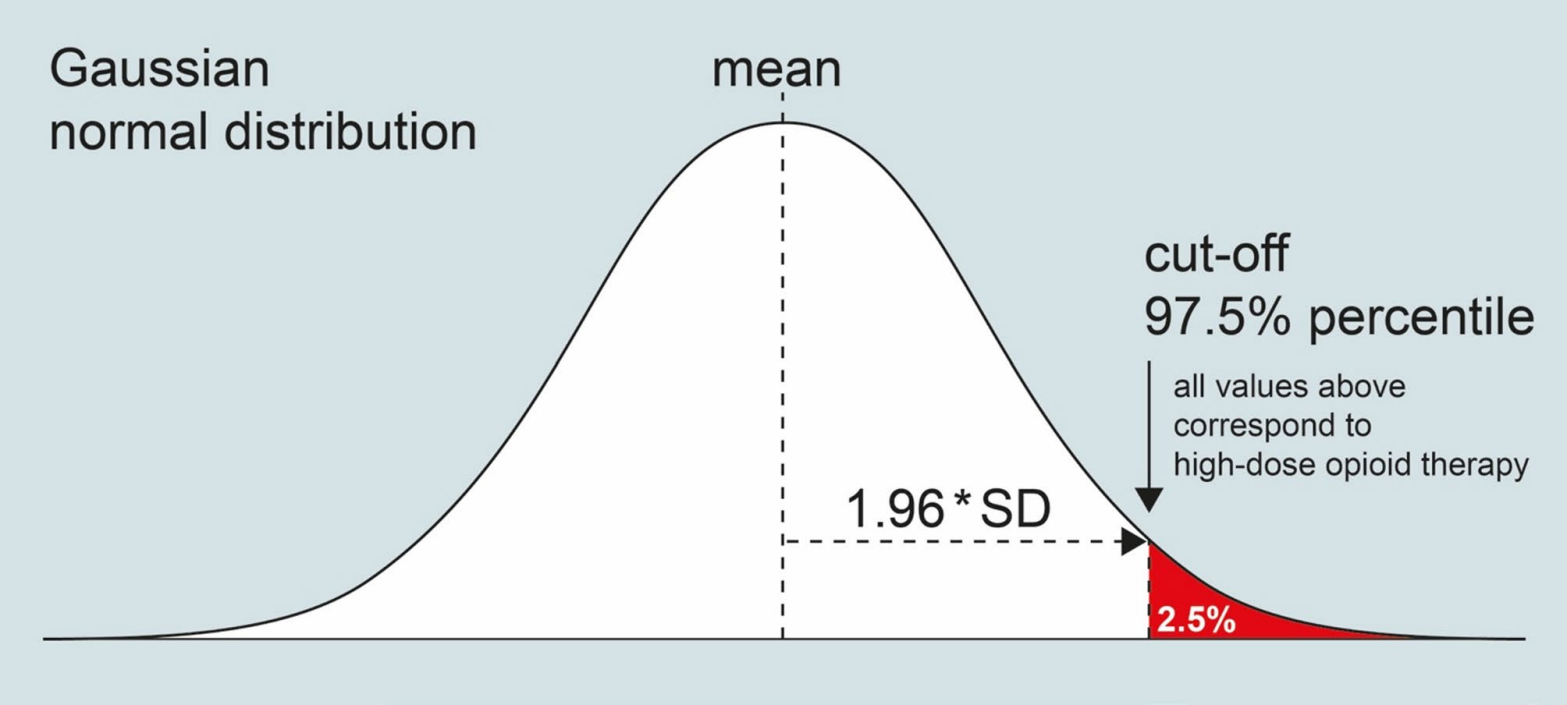
Based on a gaussian normal distribution, the 97.5% percentile can be calculated by adding 1.96 * (weighted) SD to a (weighted) mean value

**Fig. 2:**
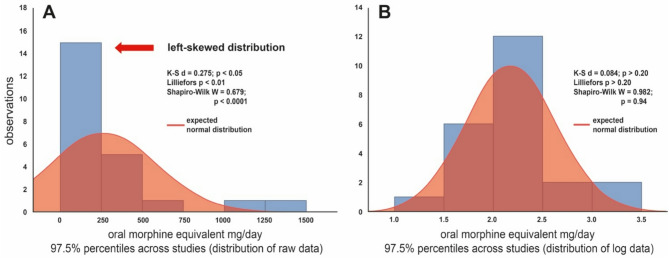
**A**. The distribution of 97.5% percentiles is left-skewed across all included studies. **B**. After log-transform data show a secondary normalization. K-S d = *Kolmogorov-Smirnov´s d*

In preparation of this meta-analysis, the heterogeneity was measured (Deeks et al. [51]) using forest plots and evaluated to decide whether the observed variation is compatible with the variation expected by chance alone (Higgins et al. [52]). Data were tested for normal distribution in Excel by examination of the descriptive statistics and based on weighted mean and SD values. A secondary normalization was performed by log transformation followed by calculation of the 97.5% percentile of the resulting data distributions (Fig. 1). The 97.5% percentile was determined as threshold for a “high-dose” opioid therapy (Fig. 2) across all chronic pain studies (23 studies with 27 study groups/arms) as well as for cancer pain (11 study groups; 1225 patients) and chronic non-cancer pain (13 study groups; 1603 patients) independently. Three study groups were not included in the subgroup analysis as both types of chronic pain were examined.

### Risk of bias

Two authors (FD, SW) independently assessed the risk of bias for each study using the assessment tools: risk of bias (RoB) 2 tool for randomized controlled trials (RCTs), ROBINS-I for interventional studies and ROBINS-E for observational studies. Non-randomized studies were assessed for seven domains of bias: allocation (bias due to confounding), selection (bias in selection of participants into the study), measurement (bias in classification of interventions), performance (bias due to deviations from intended interventions), attrition (bias due to missing data), detection (bias in measurement of outcomes) and outcome reporting (bias in selection of the reported result) (Sterne et al. [53]). RCTs were assessed in an adapted manner according to the characteristics of this study type for bias arising from the randomization process, performance bias, attrition, detection and outcome reporting bias.

In questions for which no information was available (NI) or that were not applicable (NA), comments were provided in the assessment form for justification. For each bias domain the studies were rated to be at high, moderate or low risk of bias. An overall bias was calculated in adjustment to the algorithm of the RoB 2 tool. Any disagreements were solved by discussion.

## Results

### Study selection

**Fig. 3:**
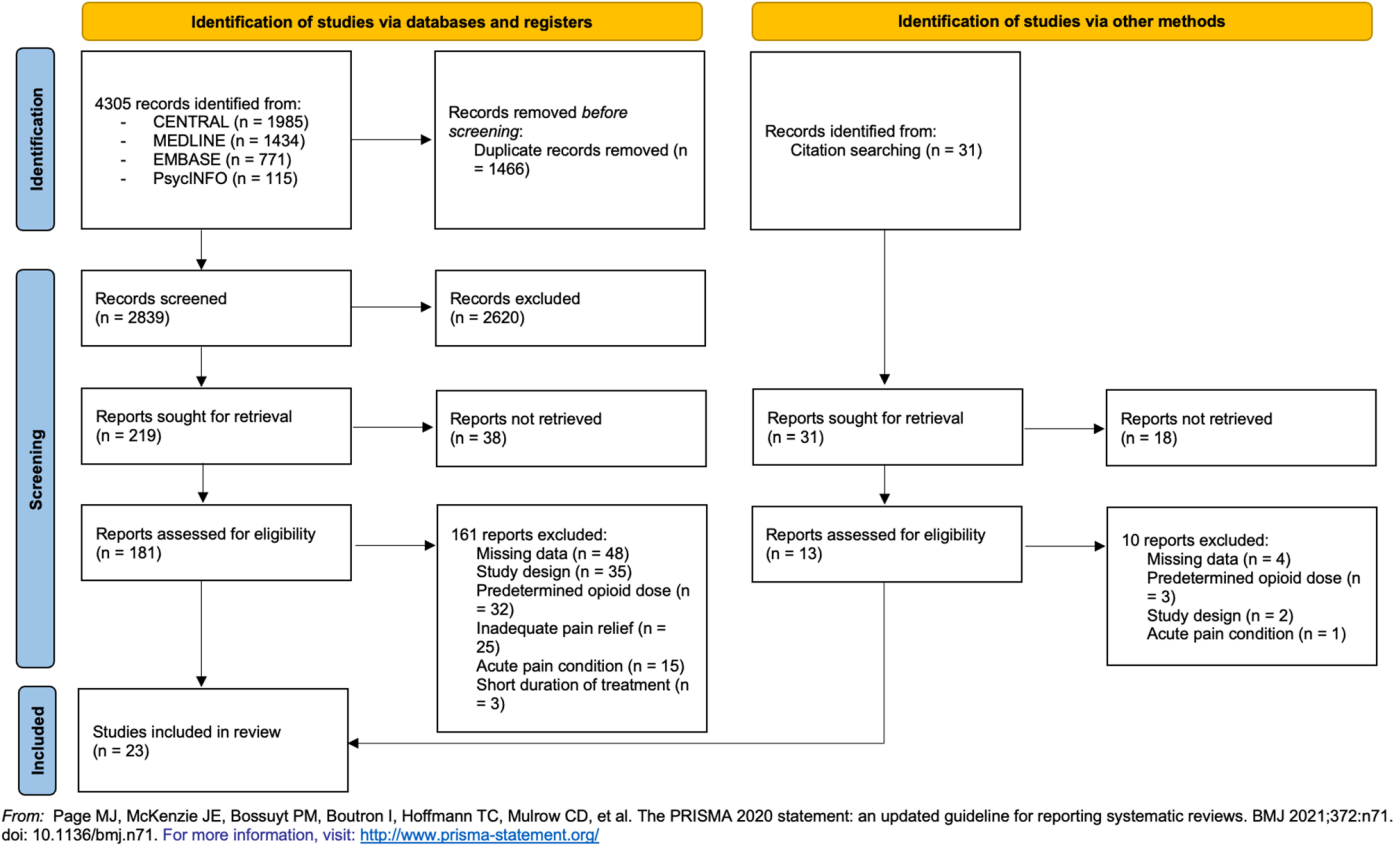
PRISMA 2020 diagram

The initial search resulted in a total of 4305 studies from 4 databases and 31 by citation searching. 1466 of these were duplicates. After exclusion through abstract screening, 219 records remained for full-text screening and 181 assessed for eligibility. None of the authors contacted for missing full-text articles responded. 161 studies were excluded for different reasons given in detail in the PRISMA flow diagram (Page et al. [40]) of the decision-making process (Fig. 3). Of the authors contacted concerning missing data, none could provide additional information. 23 studies containing 27 study groups (treatment arms) were included in the review.

Five studies were included despite the fact they mentioned an upper titration limit. Those studies investigated tapentadol and oxycodone/naloxone and the limit corresponded to the approved maximum daily dose. Studies in which the titrated dose reached the maximum were excluded.

### Study characteristics

The studies were geographically distributed with the majority conducted in European countries (8 in Italy, 3 in Germany, 2 in Switzerland and 1 in Austria). Three studies were from the Asian Region, one study from North America and one from South America. The date of publication covered the period from 1990 to 2020. Eleven were observational studies, 6 were conducted as interventional studies and 2 studies were RCTs. The treatment duration ranged from 3 to 120 days with a mean of 45 days.

In the entirety of all studies, 3111 participants were examined, 1051 (34%) of whom were located in one large study (Gatti et al. [54]). 1791 (57%) were female, the average age was 64 years. Most participants were allocated in treatment groups, in one study the participants were assigned to the control group. The underlying pain conditions were distributed into 11 study groups on CP and 13 on CNCP, three study groups investigated both entities. Participants were in equal proportions opioid-experienced (46%) and opioid-naive, at least regarding strong (WHO step III) opioids (45%). Not all studies distinguished between these groups (9%).

Eight different opioids were compared. The opioids administered orally were oxycodone or oxycodone/naloxone in 7 study groups, tapentadol (5), morphine (3), hydromorphone (3), tramadol (1) and dihydrocodeine (1). Fentanyl (2) and buprenorphine (1) were given as transdermal preparations (TD). In most studies, opioids were administered according to a fixed time schedule, that was every 12 h for oral preparations in all studies except two and every 72 h for transdermal systems. Preparations of sustained, controlled, prolonged and extended release were examined. Maintenance of existing concomitant non-opioid medication was allowed in 10 studies.

Baseline pain ranged from 4 to 8.3 on a 0 to 10 numeric rating scale with a median of 6.1 points. At the end of the study period, the median pain score was 2.2 points, which corresponds to an average reduction of 3.9 points (NRS; 0 to 10; “0”=no pain; “10”=most intense pain imaginable).

Table 1 summarizes the main characteristics of included studies.

**Table 1:** Characteristics of studies included

study	study design	country	sample size	duration	type of pain	opioid	mean opioid dose [mg]	OME per day [mg]	SD OME [mg]
Alon 2010	observational	Switzerland	144	43	both	hydromorphone	18.50	92.50	-
Aurilio 2019	observational	Italy	20	90	CNCP	tapentadol	176.00	70.40	-
Baaklini 2017	RCT	Brazil	20	28	cancer pain	morphine	103.60	103.60	59.60
Billeci 2017	observational	Italy	44	84	CNCP	tapentadol	204.50	81.80	41.12
Cascella 2019	observational	Italy	80	70	cancer pain	tapentadol	365.00	146.00	54.84
Chu 2012	RCT	United States	69	31	CNCP	morphine	78.30	78.30	37.50
Clemens 2007	observational	Germany	33	13	cancer pain	morphine	104.70	127.38	89.00
Clemens 2007	observational	Germany	44	13	cancer pain	hydromorphone	37.00	337.62	170.50
Freo 2020	observational	Italy	55	120	CNCP	tapentadol	268.90	107.56	24.76
Freo 2020	observational	Italy	59	120	CNCP	tapentadol	260.80	104.32	42.20
Gatti 2013	observational	Italy	567	60	CNCP	oxycodone/naloxone	22.20	33.30	14.70
Gatti 2013	observational	Italy	484	60	CNCP	oxycodone/naloxone	29.10	43.65	21.75
Gianni 2011	observational	Italy	93	90	CNCP	buprenorphine transdermal	34.20	75.24	-
Grond 1997	interventional	Germany	42	66	cancer pain	fentanyl transdermal	200.00	545.30	399.64
Guerriero 2015	observational	Italy	52	28	CNCP	oxycodone/naloxone	14.10	21.15	7.80
Korte 1996	interventional	Switzerland	39	28	cancer pain	fentanyl transdermal	138.00	379.60	344.40
Lazarus 1990	interventional	United States	70	21	cancer pain	morphine	126.00	126.00	11.74
Lazzari 2016	observational	Italy	186	60	CNCP	oxycodone/naloxone	21.50	32.25	14.55
Likar 2008	interventional	Austria	55	28	both	buprenorphine transdermal	41.70	98.44	8.14
Park 2016	observational	Korea	553	28	cancer pain	hydromorphone	14.50	72.50	93.50
Samolsky Dekel 2015	observational	Italy	30	90	CNCP	tapentadol	193.30	77.32	29.60
Suzuki 2008	interventional	Japan	37	20	cancer pain	oxycodone	18.92	28.38	7.85
Tessaro 2010	interventional	Italy	297	28	both	oxycodone	25.91	38.87	32.24
Wilder-Smith 2001	RCT	Switzerland	28	28	CNCP	tramadol	203.00	40.60	0.75
Wilder-Smith 2001	RCT	Switzerland	29	28	CNCP	dihydrocodeine	130.00	19.50	0.49
Wirz 2006	observational	Germany	50	14	cancer pain	hydromorphone	27.50	137.50	117.00
Zhao 2020	observational	China	257	3	cancer pain	oxycodone	55.20	82.80	114.90

### OME and opioid dose

The applied conversion rates (ranges when appropriate, see 2.4 data synthesis) for calculation of the mean OME dose were (mg, when not stated in other units):

- buprenorphine µg/h: morphine mg/d 1 : 2.2.
- dihydrocodeine : morphine 1 : 0.15 (1 : 0.25).
- fentanyl µg/h: morphine mg/d 1 : 2.7 (1 : 2.4).
- hydromorphone : morphine 1 : 5.
- oxycodone : morphine 1 : 1.5.
- tapentadol : morphine 1 : 0.4 (1 : 0.3).
- tramadol : morphine 1 : 0.2.

The weighted mean OME per day of all studies was 74.7 mg^1^ (± 55.6 mg).

The weighted mean OME between different opioids varied significantly. The opioid administered at the highest mean dose was fentanyl at 465.5 mg (± 373.0 mg) OME per day (equivalent to 172.4 µg/h fentanyl) and the lowest dispensed was dihydrocodeine at a mean dose of 19.5 mg (± 0.5 mg) OME (equivalent to 130 mg dihydrocodeine) within the studies included. Due to two outliers in the calculation a mismatch between expected and calculated distribution appeared (see Fig. 1).

Differences were also found across “opioid-naive” (47.6 ± 22.7 mg OME per day) and “opioid-experienced” groups (89.8 ± 85.3 mg) as well as between participants with an average age of less than or over 65 years (107.6 ± 93.9 mg vs. 49.2 ± 25.9 mg). Opioid-naive participants and those older than 65 years received lower doses.

When the studies were divided by means of baseline pain into 7 or more points on the NRS scale and fewer than 7 points, they also showed significant differences (48.9 ± 22.1 mg vs. 108.3 ± 99.2 mg). The same applied to distinction in terms of pain relief achieved more or less than 4.5 points on the NRS (0 to 10) scale (41.8 ± 21.1 mg vs. 113.4 ± 96.1 mg). Participants attaining less pain relief received higher doses of medication.

### Meta-analysis

The overall 97.5% percentile was determined and corresponded to 138 mg OME per day. 97.5% of the studied population received a mean OME dose below this threshold, 2.5% received a higher dose.

This dose is equivalent to oral or transdermal doses:

- ~ 140 mg/d morphine.
- ~ 65 µg/h buprenorphine transdermal.
- ~ 925 mg/d dihydrocodeine.
- ~ 50 µg/h fentanyl transdermal.
- ~ 30 mg/d hydromorphone.
- ~ 90 mg/d oxycodone.
- ~ 350 mg/d tapentadol.
- ~ 695 mg tramadol.

### Risk of bias

The risk of bias assessment revealed a low risk of bias for most of the studies in the large part of bias domains. An item with moderate or high risk for most studies was allocation bias. This contributed to a high risk of overall bias for most studies (70%).

Confounding domains preliminary considered were age, sex, severity of pain condition, co-morbidities and previous opioid therapy. Co-interventions considered relevant were other medications received during the study period. Age, sex and severity of pain condition were reported for all studies, information about previous opioid therapy was given in 17 studies (89%), yet only one provided information if appropriate methods to control for measured confounders were used.

Nearly all studies were assessed to be at low risk of selection and outcome reporting bias. The participants eligible for the trials were included and start of follow-up and intervention coincided. The reported results corresponded to the intended outcomes.

Detection bias was found to be at moderate risk for most studies as the interventional studies were open-label trials and the outcome assessors of those observational studies that provided information about this regard were aware of the intervention received.

In addition, 8 studies stated that they were at least partially funded by pharmaceutical companies, 3 of them declared no conflict of interests because the funder played no important role.

## Discussion

### Main findings

As far as we know, this is the first study to conduct a systematic approach in the identification of a “high dose” opioid therapy. The threshold before entering a high-dose opioid therapy range may serve as a useful reference point to critically reconsider the therapeutic approach. Of course, in individual patients, it remains appropriate to reach higher opioid doses following careful titration. Potential reasons include poor opioid responsiveness even after opioid rotation. The value of our analysis lies in its simple mathematical approach, which enables the description of threshold values that may provide helpful guidance, for instance, in the development of clinical practice guidelines as well as in the individual clinical use. The included studies allowed patient self-titration to satisfactory pain control, thereby offering a solid reference for patients with cancer pain (CP) and chronic non-cancer pain (CNCP). Across both groups, a 97.5th percentile threshold of approximately 138 mg oral morphine equivalent (OME) per day could be defined. When accounting for the heterogeneity of conversion factors from other opioids to morphine, this corresponds to a range of 134–139 mg/d OME. Notably, CNCP patients demonstrated a significantly lower cut-off of just under 80 mg/d OME, whereas CP patients showed significantly higher values about 290 mg/d OME.

The limits proposed here should by no means be interpreted as discouraging physicians from titrating into these ranges for individual patients. Our intention is to raise awareness that, when doing so, physicians and their patients enter the upper 2.5% of the distribution observed in other patients, where the balance between efficacy and adverse effects requires particularly careful consideration.

### Assets and constraints

The review followed the criteria of the PRISMA statement and prior to the literature search a protocol was registered. The search strategy was designed as widespread and sensitive as possible but was limited to 4 databases and papers written in English or German. Furthermore, access to free full texts was a frequent barrier. It cannot be ruled out that some studies containing relevant data were not identified. Grey literature was investigated incompletely because necessary data could not be fully extracted. Further studies were expected to be unlikely to modify the findings, however this may result in an incomplete picture.

Eligibility criteria, by their nature, are a relevant limitation for reviews. Research on the question on how to define sufficient pain relief revealed that different terms are used to describe if pain is controlled or mitigated. As Breivik et al. found, not only must there be a sufficient intensity of baseline pain to detect meaningful treatment effects, but in chronic pain, the perception and interpretation also vary over time (Breivik et al. [55]). Furthermore, the interpretation of absolute and relative pain reduction is not always consistent regarding a minimum clinical relevance (Olsen et al. [56]). Adequate pain rating on a one-dimensional scale is nearly impossible since pain is a multi-dimensional and subjective sensation that is influenced by physical, psychological, social, and spiritual factors (Mehta & Chan [57]). The transfer to a methodologically comprehensive scale has not been successful yet (Farrar et al. [58]; Olsen et al. [56]). Beside the lack of consistent terminology being used, the term “pain relief” may imply that all patients can achieve adequate analgesia through opioids. Concurrently, the term “non-responder to opioids” is found in the literature (Corli et al. [59]), which leads to the question whether the use of increasingly higher doses, if such patients do exist, is appropriate or tantamount to abuse. The inclusion criteria of a minimum level of pain relief and unrestricted titration should have reduced the risk of incorrectly low or high doses as far as possible. Nevertheless, the included studies did not mention whether titration was only upward until stabilisation or whether the dose could also be decreased over time.

Assessment of pain intensity in chronic compared with acute pain is further complicated by the appropriate timing of the survey. Like Breivik et al. stated, the “memory of pain is not accurate and often coloured by changing context factors”, therefore the “worst, least, or average pain over the last 24 h, or during the last week” may differ drastically (Breivik et al. [55]). However, due to the assessment of the pain score at multiple time points and since each was responded to with titration, this should not have affected the overall result.

Limitations regarding the quality of included studies should also be considered. The risk of bias assessment identified an overall moderate to high risk of bias due to missing information about the methodologic approach, which could not be remedied by contacting the authors wherever possible. Although poor report of methods has potential to bias the findings of included studies, it was considered the overall conclusions obtained in this review are unlikely to be affected. The type of study design also plays an important role in this context. The nature of pain therapy complicates the conduction of randomized clinical trials with a control group receiving placebo. The question arises whether observational studies generally have a high risk of bias because of their retrospective view and sometimes inconsistent methodology (Nguyen et al. [60]).

One of the major problems emerged from the determination of an oral morphine equivalent. The included studies investigated different opioids, both classified as strong (WHO step III) or weak (WHO step II). Many meta-analyses have been undertaken summarizing the inconsistently used conversion rates for various opioids (Nielsen et al. [47]; Treillet et al. [61]). However, it is noticeable that there are a vast number and even globally different recommendations (Dowell et al. [46]; Häuser et al. [20]). Since consideration must be given to opioid experience, switching between opioids, and routes of administration, it was not possible to clearly determine which source was to be preferred. By comparing a variety of sources, a conversion rate as universal as possible should have emerged for each opioid.

In this context, it is also important to illuminate the differences in pharmacodynamics. Buprenorphine shows high affinity but is a partial agonist for the µ-opioid receptor. Therefore, it can hardly be antagonized by naloxone, but due to its ceiling effect at the same time shows lower rates of side effects such as respiratory depression (Davis et al. [62]). Tapentadol and tramadol meanwhile show a dual mechanism of action; both inhibit the reuptake of noradrenaline and thereby modulate descending inhibitory pain pathways in the spinal cord, with tapentadol exhibiting a predominantly noradrenergic and tramadol a more serotonergic component (Tzschentke et al. [63]). Hence, the evidence for equianalgesic tables to compare opioids is an ongoing discussion (Treillet et al. [61]).

It is essential to consider the heterogeneity of included studies. Even though a meta-analysis was feasible according to the statistical calculation, the variety of chronic pain conditions and types of pain might influence the valuation of a joint threshold. Subgroup analysis revealed significant differences in the dosage for cancer pain and chronic non-cancer pain. This is unsurprising considering the underlying differences. While cancer pain has an identifiable underlying cause and can therefore be clearly classified as chronic secondary pain, musculoskeletal pain can occur both primary and secondary (Nicholas et al. [64]). A large proportion of studies on CNCP examined chronic low back pain (CLBP), but not all have been more specific about whether an underlying cause such as osteoarthritis could be identified. This finding may indicate that a separate threshold for CNCP and cancer pain would be useful, however, this is contrasted with the rationale that this model only can provide a recommendation for further clinical investigation.

This review aimed to describe high-dose ranges of opioid therapy in chronic pain, focusing on doses beyond the 97.5th percentile cut-off identified in the analysed studies. This raises the question of whether the selected studies can be considered a representative collective. Even though the worldwide distribution of studies was not homogeneous and some of the studies were older, the transferability of the results was attempted by developing the eligibility criteria.

The possibility of a sex difference in pain perception and opioid analgesia must also be considered when applying a uniform threshold. Numerous studies have found an increased pain sensitivity in women (Bartley & Fillingim [65]). This could lead to the conclusion that, on average, women may also need higher doses of opioids. Unfortunately, the findings for this assumption are inconsistent (Bartley & Fillingim [65]; Niesters et al. [66]) and none of the included studies gathered opioid doses for the sexes separately, therefore the need for different thresholds in women and men cannot be ruled out. However, these considerations point to the need of sex sensible study designs.

It must also be considered that other symptoms, such as anxiety or other affective symptoms, might influence treatment to higher doses. Through the binding of ∂-opioid receptors, opioids have an anxiety-relieving effect, which could be particularly influential in critical illnesses such as advanced cancer up to palliative situations. Maybe due to a blind spot to psychological factors, in most studies there was no information about comorbidities or psychosocial symptom burden as a main impact of pain (Taylor et al. [67]; Turk et al. [68]). Although studies with indications other than pain were excluded, the influence of coexisting symptoms cannot be ruled out.

Another factor influencing the required opioid dose could include the mean duration of illness and opioid-naivety. If expectations regarding therapy effectiveness are high, the placebo-effect may also play a role. While the definition limit for chronic pain is at least 3 months (WHO [11]), many patients have suffered from pain for years and tried various medications during that time. Because opioids lead to tolerance development over time, a longer duration of illness may be associated with the need for higher doses. In addition, a selection of patients who already have a longer history of suffering cannot be ruled out, as they may be more willing to participate in studies. Unfortunately, it was not possible to extract the mean duration of illness from all studies to perform a subgroup analysis. Previous pain medication in the examined collective included both non-opioids and opioids. Here, a distinction into subgroups was possible for most studies and it could be concluded that opioid-experienced patients require significantly higher doses. This fact should be considered when applying a cut-off. However, the influence of a switch in opioids could not be analysed.

Chronic pain is often mixed-pain and opioids are critically discussed especially for the treatment of neuropathic pain. Co-medication such as anticonvulsants, which were allowed in some studies and are recommended in the neuropathic pain guideline ahead of opioids, may have affected the opioid dose (Häuser et al. [20]; Nadeau & Lawhern [33]).

There are numerous other factors for which an influence on the metabolism of opioids have been described, such as the influence of gene variants (Lotsch et al. [69]), CYP enzymes (Crews et al. [70]) and chronic inflammation, for example due to smoking (Zevin & Benowitz [71]). They may lead to a larger opioid consumption as well as opioid-induced hyperalgesia (Bannister & Dickenson [72]). Consideration of all possible factors was beyond the scope of this review but should be further explored in future studies.

### Future directions

The threshold identified in this meta-analysis could provide guidance for physicians prescribing opioids for chronic pain patients. This, in turn, could increase drug safety because the comparison to commonly prescribed dosages has been simplified. The identified high-dose thresholds should be reflected and confirmed from a clinical perspective by future studies. Reported high-dose ranges are not intended to restrict individualized decisions that balance efficacy with adverse effects within the patient’s biopsychosocial and spiritual context.

## Supplementary Information

Below is the link to the electronic supplementary material.

**Supplementary Material 1**: **Appendix**: PRISMA 2020 checklist. Search strings.

## Data Availability

The datasets are available from the corresponding author on reasonable request. The search strings can be found in the appendix and on searchRxiv https://searchrxiv.org/.

## Footnotes

1 Note: All miligram figures are rounded to whole numbers with the first decimal place.

## Abbreviations

CNCP: Chronic non-cancer pain
CP: Cancer pain
NRS: Numeric rating scale
OME: Oral morphine equivalent
SD: Standard deviation
TD: Transdermal preparations
